# An evaluation of predictors for success of two-duct ligation for drooling in neurodisabilities

**DOI:** 10.1007/s00415-020-09735-1

**Published:** 2020-02-06

**Authors:** Stijn Bekkers, Karen van Hulst, Corrie E. Erasmus, Corinne P. Delsing, Arthur R. T. Scheffer, Frank J. A. van den Hoogen

**Affiliations:** 1 Department of Otorhinolaryngology and Head and Neck Surgery, Radboud University Medical Centre, Philips Van Leijdenlaan 15 (route 377), Postbus 9101, Nijmegen, 6500 HB The Netherlands; 2 Department of Rehabilitation, Donders Institute for Brain, Cognition and Behaviour, Nijmegen, The Netherlands; 3 Department of Pediatric Neurology, Donders Institute for Brain, Cognition and Behaviour, Nijmegen, The Netherlands

**Keywords:** Spasticity, Drooling, Oromotor function, Posture

## Abstract

**Background:**

Drooling is dependent on various clinical variables. However, while drooling proves refractory to two-duct ligation in 40% of patients, predictors for treatment success are sparse and to date there is little evidence why some respond well while others are non-responders. We aim to find predictors for treatment success and study the effectiveness of two-duct ligation for drooling in neurodisabilities.

**Methods:**

Fifty-four patients with moderate to severe drooling who had undergone two-duct ligation were screened for inclusion. Four patients were excluded due to missing or unreliable primary outcomes. The average age at the time of surgery was 12 years. Predictors were evaluated for treatment success which was defined as ≥ 50% visual analog scale for severity of drooling and/or drooling quotient reduction from baseline. Treatment effect was measured after 8 and 32 weeks compared to baseline.

**Results:**

Age (more mature), adequate posture (no anteflexion), and normal speech are predictors for treatment success. Compared to baseline, drooling quotient was significantly lower at 8 (difference 18.6%, 95% confidence interval 12.3–24.9%) and 32 weeks (difference 10.1%, 95% confidence interval 3.9–16.4%). Compared to baseline, visual analog scale was significantly lower at 8 (difference 45.0, 95% confidence interval 37.0–52.9) and 32 weeks (difference 32.9, 95% confidence interval 25.0–40.7).

**Conclusions:**

Age, adequate posture, and a normal speech are predictors for treatment success, are easily determined pre-operatively, and help the clinician providing patient-specific probability of treatment success. There is a significant subjective and objective decrease of drooling after two-duct ligation.

## Introduction

When drooling in children with neurodisabilities is not adequately managed with conservative treatment or botulinum neurotoxin A (BoNT-A), and age progresses, surgery is advocated in our institution [1].

In cases of isolated anterior drooling (visible drooling), the treatment of choice is submandibular duct rerouting (SMDR). Although SMDR is effective for > 80% of patients, it is an extensive procedure with approximately 8% risk for serious adverse events (SAE) and in one cohort studied a mean of 4.4 days admission [2].

Submandibular gland excision (SMGE) is commonly advised when SMDR is contraindicated. Although the response to treatment is good, the risk for nerve damage, an external scar, and longer admission and surgical/anesthetic time make the procedure unappealing [3–6].

2-DL was recently presented as an alternative procedure that is effective in > 60% of patients with specific advantages over SMDR and SMGE [7]. 2-DL is a more limited and shorter procedure that can be used for both anterior and posterior drooling. There is no external scar and limited SAE. Unfortunately, drooling proves refractory to 2-DL in approximately 40% of children [8].

Drooling is multifactorial and dependent on various clinical variables (poor gross motor function, dental malocclusion, poor posture, etc.) [9–11]. However, thus far, clinical variables predicting treatment failure are sparse [2, 9], so current surgical decision making is based on either age, contraindications for SMDR or expert opinion. Moreover, to date it remains unrevealed why some patients respond well to surgery while some recur and others are non-responders. For this study, we aim to find the predictors for treatment success of 2-DL.

## Method

### Study design

Patients who had undergone 2-DL to reduce anterior drooling were retrospectively screened for inclusion, and the primary aim of the study was to evaluate predictors of 2-DL treatment success. All patients were seen in the outpatient saliva control clinic at the Radboud University Nijmegen Medical Centre where patient characteristics and measurements were prospectively obtained by specifically trained speech and language therapists (SLTs).

### Participants

Patients aged 8 years or older, with cerebral palsy or another non-progressive neurodevelopmental disability, and anterior drooling treated with 2-DL between July 2006 and December 2017 were eligible for inclusion. The exclusion criteria were drooling severity (never (1), mild (2), moderate (3), severe (4), profuse (5); DS) score ≤ 2 in combination with drooling frequency (no (1), occasional (2), frequent (3), constant (4); DF) score ≤ 1 indicating mild or absent drooling, recent (< 6 months) glandular botulinum neurotoxin A (BoNT-A) injection, and simultaneous alternative treatment for drooling. Furthermore, we excluded patients with missing values for both visual analog scale (VAS) and drooling quotient (DQ) at baseline or 32 weeks follow-up (because it was not possible to calculate 32 weeks treatment success). Patients were excluded from the primary analyses when there were missing values for the dependent or independent variables. We did not impute missing data. The study was conducted in accordance with the national and international ethical standards laid down in the 1964 Declaration of Helsinki and its later amendments. The regional committee on Research Involving Human Subjects approved the study. Caregivers’ informed consent was given before each intervention.

### Procedures

2-DL was performed under general anesthesia in an outpatient setting. At the beginning of the procedure, the floor of the mouth was infiltrated with 1% lidocaine with 1:100.000 epinephrine. Subsequently, the floor of the mouth was incised parallel to the frenulum, and the submandibular duct was identified on both sides and exposed for approximately 1.5 cm. After sufficient exposure, the ducts were ligated using a disposable stapler applying two vascular clips per duct. Finally, the floor of mouth incision was closed using absorbable sutures. Patients received amoxicillin/clavulanic acid for 7 days, and paracetamol and diclofenac for 5 days post-operatively.

### Outcome measures

Measurements, assessing the objective and subjective outcomes, were made prior to surgery and 8 and 32 weeks after treatment. Specific clinical variables (e.g*.* posture, dental occlusion, oral motor functions, degree of disability) were gathered during the initial intake at the drooling clinic. Clinical variables and other patient characteristics were obtained from the medical records.

### Hypothesis

Based on a thorough literature search and earlier studies of the same research group, the following six factors were hypothesized to contribute most to drooling severity: age, head posture, dental occlusion, tongue protrusion, control of voluntary movement functions, very severe speech disorder (VSSD) defined as either no speech, or anarthria or very severe dysarthria according to the Therapy Outcome Measure for Dysarthria (TOM-Dysarthria) [2, 3, 9, 10, 12]. A VSSD is either due to impaired oromotor function (dysarthria), or due to insufficient cognitive capabilities. Measurements were obtained by specialized SLTs.

### Primary outcomes

The primary aim of the study was to evaluate predictors of 2-DL treatment success, defined as ≥ 50% reduction in VAS and/or DQ from baseline to 32 weeks. VAS for severity of drooling during the past 2 weeks was rated by parents or caregivers on a line ranging from 0 (no drooling) to 100 (severe drooling) during each visit at the drooling clinic. The DQ is a validated, semi-quantitative observational outcome for measuring new saliva dripping over the lips during 15 s intervals, in a 5 min standardized setting [13]. In this study we report the DQ which was measured in activity [14]. Patients were evaluated while awake and sitting erect, at least 1 h after eating.

### Secondary outcomes

Secondary outcomes were:

1. VAS for severity of drooling from baseline to 32 weeks.
2. DQ from baseline to 32 weeks.
3. DS and DF from baseline to 32 weeks.
4. Surgical time.
5. Adverse events (AEs). AEs were graded as related or unrelated to the surgical procedure where pain, dysphagia and xerostomia for less than 7 days were considered normal post-operative course [8, 15]. AE that were potentially life threatening, required prolonged hospitalization, surgical re-intervention, or caused permanent damage are defined as SAE.
6. To evaluate whether predictors for objective treatment success (defined as a ≥ 50% reduction in DQ) are similar to predictors for combined treatment success (defined as a ≥ 50% reduction in DQ and/or VAS). Objective treatment success (≥ 50% reduction in DQ from baseline to 32 weeks) served as the primary outcome in previous prediction studies of the same research group.

### Statistical analyses

Clinical variables that are potentially related to treatment success were used in the logistic regression. Logistic regression statistics was used to analyze predictors to treatment success using backward selection. We present a prediction model for the primary outcome. Discrimination is presented as bias-corrected area under the curve (AUC). Internal validation was performed by the bootstrap method (1000 bootstraps). We present the calibration per quintiles as a figure. Goodness of fit was evaluated using a Hosmer–Lemeshow test for quintiles and significance was set at ≤ 0.05 which would indicate a poor fit. We did not perform an external validation.

The effect of 2-DL was analyzed by comparing baseline DQ, VAS, DS and DF scores to the scores at 32 weeks. Paired sample *t* tests were used for the continuous variables (VAS and DQ), and a Wilcoxon rank test was used for the categorical variables (DS and DF, Table 3). The level of significance was set at ≤ 0.05 except for the logistical regression analyses where the level of significance was set at ≤ 0.25 because of the small sample size of the cohort. We present odds ratios and 95% confidence intervals (CIs).

## Results

Fifty-four patients were screened for eligibility. One patient was excluded due to missing baseline primary outcome data and two patients were excluded due to missing primary outcome data at 32 weeks follow-up. One patient was excluded because of a bilateral sublingual gland excision following bilateral ranula formation (SAE), because surgery interfered with salivary secretion and therewith reliable evaluation of 2-DL effect. This patient was not excluded from the AE analyses. Ten patients were excluded from the primary analyses (logistic regression) due to missing independent variables. Forty patients were left for logistic regression analyses. The mean age at surgery was 12.1 years (standard deviation [SD] = 3.5, ranging 8 to 23 years old), the mean interval between baseline measurement and surgery was 143.4 days (SD = 115.9), and the predominant main diagnosis was cerebral palsy (58%) versus other neurodevelopmental disabilities (genetic [no craniofacial], syndromic, not specified disorders) (42%). Additional patient characteristics are presented in Table 1.

**Table 1:** Patient characteristics (*n* = 50)

Age, years, mean ± SD	12.1 ± 3.5
Female sex, *n* (%)	
Male	22 (44.0)
Female	28 (56.0)
Main diagnosis, *n* (%)	
Cerebral palsy	29 (58.0)
Other neurodevelopmental disability	21 (42.0)
Degree of disability, *n* (%)	
Ambulant	16 (32.0)
Non-ambulant	34 (68.0)
Developmental age, *n* (%)	
< 4 years	33 (66.0)
> 4 years	17 (34.0)
Epilepsy, *n* (%)	
Controlled	26 (52.0)
Intractable	6 (12.0)
No	18 (36.0)
Very severe speech disorder (VSSD), *n* (%)	
Yes	32 (64.0)
No	18 (36.0)
Poor posture (anteflexion), *n* (%)	
Yes	21 (42.0)
No	24 (48.0)
Missing	5 (10.0)
Tongue protrusion, *n* (%)	
Permanent–often	22 (44.0)
Sometimes–never	24 (48.0)
Missing	4 (8.0)

*Non-ambulatory status* Gross Motor Function Classification System score IV–V; *VSSD* very severe speech disorder defined as no speech, anarthria or very severe dysarthria vs. severe–moderate–mild–no dysarthria, Tongue protrusion: Permanent–often vs. sometimes–never

### Prediction model for treatment success

Treatment success, defined as a ≥ 50% VAS and/or DQ reduction from baseline to 32 weeks, was reached in 24 patients (60.0%, *n* = 40). Analogous to our earlier studies, variables potentially related to treatment success were analyzed in a logistic regression. Based on previous literature, six variables were entered in the logistic regression analyses. Univariate analyses showed no multicollinearity between the included variables. Univariate analyses showed that patients with a poor posture (anteflexion) profit least from 2-DL (Table 2).

**Table 2:** Prediction model (*n* = 40)

Clinical characteristics	Univariable analyses		Multivariable analyses	
	*r*	*p* value	Adjusted OR (95% CI)	*p* value
Age (continuous)	0.30	0.063*	1.25	0.099*
Non-ambulant	0	1.0		
Anteflexion	− 0.35	0.028*	0.24	0.056*
Dental malocclusion	0.16	0.32		
Tongue protrusion	− 0.14	0.38		
VSSD	− 0.19	0.25	0.39	0.22*
			Nagelkerke R_2_ = 0.29	

*p* value ≤ 0.25; *non-ambulant* Gross Motor Function Classification System score IV–V, *Anteflexion* head posture; tongue protrusion: permanent–often vs. sometimes–never; very severe speech disorder (VSSD) = no speech, anarthria or very severe dysarthria vs. severe–moderate–mild–no dysarthria

After backward selection, three factors were identified as predictors of treatment success: age, poor posture, and the presence of very severe speech disorders (VSSD).

**Fig. 1:**
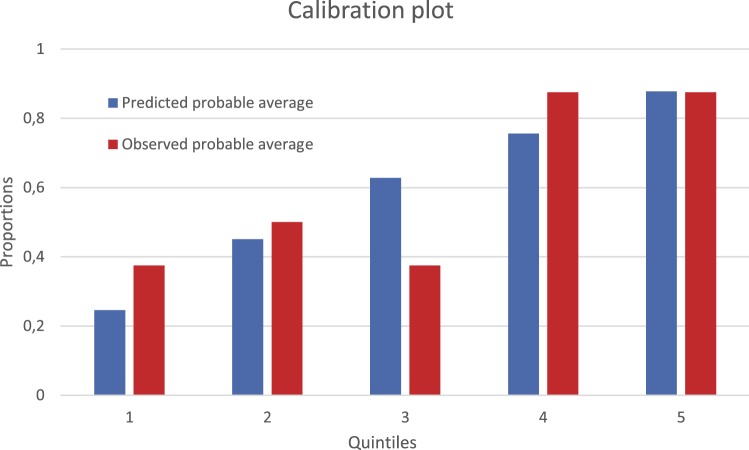
The figure represents quintiles between predicted probable average and observed probable average. The quintiles are in ascending order of probability. The figure illustrates that there is a strong agreement between the predicted and observed probability

The bias corrected AUC was 0.68. Before bias correction the AUC was 0.79. Quintiles between predicted probable average and observed probable average were closely related (Fig. 1). The figure illustrates that there is a strong agreement between predicted and observed probability which shows that there is a strong relation between the prediction model and the true data. Moreover, Hosmer–Lemeshow test indicated a good fit of the model (*p* = 0.97, *x*^2^ 0.27, *df* 3).

Based on the logistical regression analysis, the formula to calculate the probability for treatment success for 2-DL is:

$$ {\text{Odds ratio }} = \, - 0.{94 } + \, 0.{22 }*{\text{ age }}{-}{ 1}.{43 }*{\text{ anteflexion }}{-} \, 0.{95 }*{\text{ very severe speech disorder,}} $$

$$ {\text{Probability }} = {\text{ odds ratio}}/\left( {{1 }{-}{\text{ odds ratio}}} \right). $$

### Secondary outcomes

#### Treatment effect

**Fig. 2:**
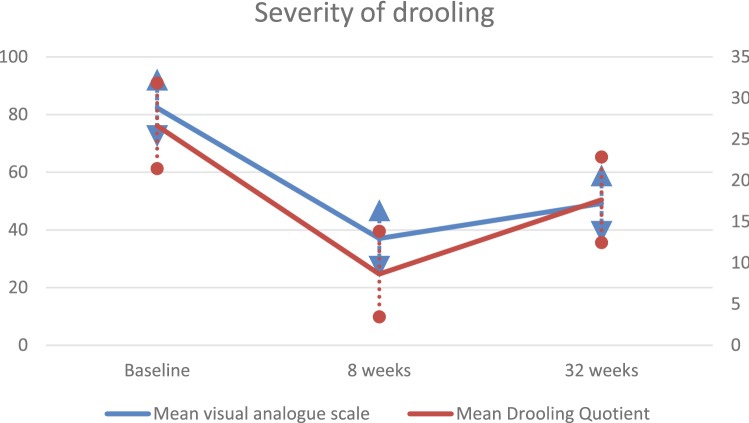
Mean visual analog scale and standard error in blue at baseline, 8 and 32 weeks follow-up, range 0–100. Three out of the total 150 VAS values were missing (2.0%). Mean drooling quotient and standard error in red at baseline, 8 and 32 weeks follow-up, range 0–30%. Eight out of the total 150 DQ values were missing (5.3%)

The treatment effect was similar to previous studies. VAS was significantly lower at 8 (difference 45.0, 95% CI 37.0–52.9) and 32 weeks (difference 32.9, 95% CI (25.0–40.7) when compared to baseline (mean VAS 82.0, SD 14.0) (Fig. 2). Similarly, the DQ was significantly lower at 8 (difference 18.6%, 95% CI 12.3–24.9%) and 32 weeks (difference 10.1%, 95% CI 3.9–16.4%) when compared to baseline (mean DQ 26.7%, SD 20.1) (Fig. 2).

#### Drooling severity and drooling frequency scale

Both DS and DF were significantly lower at 8 and 32 weeks when compared to baseline (*p* < 0.001, Table 3). Both DS and DF increased significantly from 8 to 32 weeks follow-up.

**Table 3:** Drooling severity and drooling frequency scale (*n* = 50)

DS	*n* (%)					*p*
	~ 1	~ 2	~ 3	~ 4	~ 5	
Baseline (*n* = 49)	−	-	1 (2)	13 (27)	35 (70)	
8 weeks (*n* = 48)	2 (4)	4 (8)	16 (33)	14 (29)	12 (25)	< 0.001
32 weeks (*n* = 50)	−	6 (12)	6 (12)	19 (38)	19 (38)	< 0.001

DF	‘1	‘2	‘3	‘4	
Baseline (*n* = 49)	−	4 (8)	16 (33)	29 (59)	
8 weeks (*n* = 48)	3 (6)	29 (60)	9 (19)	7 (15)	< 0.001
32 weeks (*n* = 50)	1 (2)	24 (48)	12 (24)	13 (26)	< 0.001

*pp* value, *DS*  drooling severity,  ~ 1 dry, ~ 2  mild, ~ 3 moderate, ~ 4  severe,  ~ 5 profuse, *DF*  drooling frequency, ‘1 never, ‘2 occasional, ‘3 frequent, ‘4 constant

#### Surgical time

The mean surgical time was 24 min (SD 14 min) and mean time in theater was 64 min (SD 20 min).

#### Adverse events

There were 16 AEs of which 4 were considered as serious (Table 4). Ten AE’s resolved without re-intervention. In three cases, children were readmitted due to nausea and dehydration (SAE), one child underwent surgical re-intervention due to bilateral ranula, and two patients received oral antibiotics for pneumonia.

**Table 4:** Adverse events (*n* = 51)

Total amount of AE, *n* (%)	16 (31.4%)
AE, *n* (%)	12 (24.0%)
Post-operative bleeding, *n* (%)	1 (2.0%)
Prolonged antibiotics for a floor-of-mouth cyst, *n* (%)	1 (2.0%)
Diminished feeding due to nausea, *n* (%)	1 (2.0%)
Prolonged pain medication, *n* (%)	2 (3.9%)
Antibiotics for pneumonia, *n* (%) possibly related to the intervention	2 (3.9%)
Xerostomia, *n* (%)	2 (3.9%)
Swelling of the submandibular region, *n* (%)	2 (3.9%)
Dysphagia, *n* (%)	1 (2.0%)^a^
SAE, *n* (%)	4 (7.8%)
Direct post-operative admission due to nausea, *n* (%) related to the intervention	1 (2.0%)
Admission due to nausea, *n* (%) unrelated to the intervention	1 (2.0%)^a^
Admission because of dehydration due to gastroenteritis, *n* (%)	1 (2.0%)^a^
Bilateral sublingual gland excision due to bilateral ranula, *n* (%) related to the intervention	1 (2.0%)

*2-DL* two-duct ligation, *AE* adverse event, *SAE* serious adverse event

^a^Unrelated to the intervention

#### A comparison between predictors for treatment success and objective (DQ) treatment success

The predictors associated with objective treatment success (DQ only) were identical to the ones identified for combined treatment success (VAS and/or DQ).

## Discussion

The purpose of this study was to identify predictors for treatment success. We found age (more mature), adequate posture (no anteflexion), and normal speech (no VSSD) to increase the chance of successful treatment of anterior drooling.

Approximately 40% of children show no clinical response to 2-DL. Although multiple etiological variables have been identified for drooling (dysfunctional oral motor control, and swallowing disorders preventing effective salivary swallowing, poor posture, lack of speech, lack of mouth closure, epilepsy, poor gross motor function) [9–11], it has until now been unknown which of these are predictive factors of treatment success in 2-DL. Previous studies on predictive factors for other treatments have shown variable results; a previous study on predictors for the effect of intraglandular BoNT-A revealed no significant clinical variables [9]. Another study from the same research group identified both age (younger than 12 years) and poor posture (anteflexion) to be associated with SMDR treatment failure [2]. Anteflexion as negative predictor for SMDR is logical, as anteflexion interferes with the relocation of salivary flow. Interestingly, anteflexion and age are also associated with 2-DL treatment failure.

There are many possible explanations why increasing age is associated with a higher response rate to surgery. With increasing maturity (and corresponding developmental age), patients might learn to better manage their saliva (either due to increasing awareness of swallowing or wiping of saliva), leading to improved outcomes. There could be ongoing motor development, or more mature patients might (1) be more motivated and (2) be more aware of drooling in specific, social circumstances. Another possibility is that more severely affected patients undergo surgery at a younger age. This is, however, not supported by the data. Lastly, younger patients exchange teeth which could lead to increased salivation, so this might temporarily influence treatment effect.

A VSSD, defined as no speech, anarthria or very severe dysarthria, is associated with treatment failure. VSSD occurs in patients with impaired oromotor function (anarthria or very severe dysarthria), insufficient intellect (no speech), or both (impaired oromotor function is associated with insufficient intellect [16]). In contrast, children with better speech capacities have better outcomes after 2-DL. These children have more oral motor capabilities and the cognitive level to benefit from reduced saliva. Our definition for VSSD makes it impossible to distinguish between oromotor disorders and impaired intellect as specific predictive factors for treatment failure. Nevertheless, speech is an easily definable clinical characteristic.

In summary, age, poor posture, and a VSSD are simply determined in a multidisciplinary, pre-operative setting. The formula to calculate the probability of 2-DL treatment success is helpful when providing patient-specific pre-operative information. Clinicians should be attentive to the modest individual effect sizes of the clinical variables. Furthermore, when considering this vulnerable patient population, the predictors inform, but never replace individual care. Moreover, because the data were not externally validated, clinicians in other institutions should be attentive to patient’s specific history when using the formula for surgical decision making. Additionally, we advocate that drooling should be treated in a multidisciplinary approach that includes a (child) neurologist, ENT surgeon, and a speech and language therapist who evaluates speech but also other aspects of oral motor function.

We identified the same predictors for both definitions of treatment success (objective and ‘combined’ treatment success). We feel it is of more clinical value to evaluate the subjective as well as the objective effects of treatment. Going forward, we therefore intend to use combined treatment success as an outcome in future research [8].

2-DL seems to be an effective treatment strategy for anterior drooling up to 32 weeks (10.1% decrease to baseline) that is only slightly less effective when compared to SMDR and SMGE at 8 weeks (15.6% resp 23.6%). [2, 17] Moreover, 2-DL is a relatively short procedure (24 min surgical time vs. 93 min in SMDR) that is performed in a day case setting with low (4%) risk for SAE related to the intervention (there were only two SAE related to the intervention which included direct post-operative admission due to nausea, and surgical re-intervention due to bilateral ranula formation) compared to 8% after SMDR and SMGE [2, 17]. Future studies should investigate the position of 2-DL when compared to SMGE and SMDR.

### Strengths and limitations

The strengths of this study include the measurements of both objective and subjective outcomes that were obtained in a prospective setting. The measurements were collected at baseline and follow-up measurements so we could assess change over time and calculate treatment effect. This enabled us to identify three easily definable clinical variables that are associated with treatment success. There are, however, several limitations of the study. On the one hand, this is the largest cohort of patients that underwent 2-DL. On the other, the cohort still consists of a relatively small number of patients, limiting the amount of variables that could be entered in the logistic regression analyses. Patients were excluded due to missing 32-weeks follow-up which might induce a selection bias. This however included only two patients, so bias should be limited. Moreover, data were retrospectively collected in one institution, and we did not perform an external validation for the prediction model which might limit the extent to which the results can be generalized. However, we expect that the results might be generalizable because: (1) patients are generally comparable [2]; (2) 2-DL is a relatively easy surgical technique for which the procedure is thoroughly described in the manuscript; (3) based on the methods, age, poor posture, and a VSSD are easy to determine in other institutions.

Drooling proves refractory to 2-DL in ~ 40% of patients after 32 weeks. Age (more mature), posture (no anteflexion) and normal speech (no VSSD) are predictors for 2-DL treatment success. Clinicians could opt for a more aggressive approach (e.g. 3-duct ligation or 4-duct ligation) in case of anteflexion and a VSSD. Future studies should, however, externally validate the predictors for treatment success before the model is to be used in surgical decision making. As the effect of 2-DL is greater in more mature patients, our restraint to advise surgery for anterior drooling in younger children (~ < 10 years of age) is substantiated. Moreover, 2-DL interferes with SMDR which is currently the more successful treatment for drooling, so the indication should be carefully considered, especially when children are not yet mature. SMDR is, however, contraindicated in patients with progressive developmental disorders, a history of aspiration pneumonia, and posterior drooling (saliva aspiration) [2, 18, 19]. In these cases, 2-DL can be considered, but future studies should investigate the effect of 2-DL in the long term.

## Data Availability

The anonymous demographics and data will be shared on request from any qualified investigator.

## Abbreviations

2-DL: 2-Duct ligation
DQ: Drooling quotient
VAS: Visual analog scale
AE: Adverse event
SAE: Serious adverse event
DS: Drooling severity
DF: Drooling frequency
BoNT-A: Botulinum neurotoxin A
SMGE: Submandibular gland excision
SMDR: Submandibular duct rerouting
VSSD: Very severe speech disorder
TOM-Dysarthria: Therapy Outcome Measure for Dysarthria
AUC: Area under the curve
CI: Confidence interval
SD: Standard deviation
CP: Cerebral palsy
